# Dual-targeting nanoparticles for reprogrammed T cell responses in the tumor microenvironment

**DOI:** 10.1186/2051-1426-2-S3-P108

**Published:** 2014-11-06

**Authors:** Alyssa K Kosmides, Jonathan Schneck

**Affiliations:** 1 Johns Hopkins University, Baltimore, MD, USA; 2 Johns Hopkins School of Medicine, Department of Pathology, Institute for Cell Engineering, Baltimore, MD, USA

## 

One of the largest obstacles in cancer immunotherapy involves overcoming the immunosuppressive tumor microenvironment [1]. While many therapies are focused primarily on activating antigen-specific CD8+ T cells, the tumor microenvironment often expresses immunosuppressive cytokines and other immunoregulatory proteins such as checkpoint blockade molecules that diminish their effects [2]. Programmed death ligand 1 (PD-L1) is an inhibitory checkpoint molecule upregulated on many cancers, including melanoma, ovarian cancer, and renal cancer [3]. This can shield a tumor from immune attack by binding to its receptor, PD-1, on T cells. We have developed a nanoparticle platform that combines blockade of PD-L1 with the T cell co-stimulatory signal, anti-4-1BB. This dual targeting system redirects effector cells to recognize target cells while simultaneously blocking checkpoint inhibitors. Antagonistic anti-PD-L1 antibodies and agonistic anti-4-1BB antibodies are conjugated to the surface of biocompatible 50-100 nm iron dextran nanoparticles. The nanoparticles cause a 6-fold increase in IFN-γ production by CD8+ T cells with an exhausted phenotype in the presence of tumor cells *in vitro*. Additionally, we have shown tumor suppression and a 30% decrease of PD-1 expression in tumor infiltrating lymphocytes in an *in vivo *B16 mouse melanoma model. This approach may not only reprogram local signaling within the tumor microenvironment, but also promote polyclonal cytotoxic T cell responses in the absence of defining the antigenic specificity of the infiltrating T cells.
